# Between hope and hype: assessing artificial intelligence in cardiovascular imaging

**DOI:** 10.1093/ehjimp/qyaf149

**Published:** 2025-11-25

**Authors:** Federico Fortuni, Giuseppe Ciliberti, Fabrizio Ricci, Frontoni Emanuele, Erberto Carluccio, Antonella Moreo

**Affiliations:** Cardiology and Cardiovascular Pathophysiology, S. Maria della Misericordia Hospital, University of Perugia, Piazzale Giorgio Menghini, 3, Perugia 06129, Italy; Cardiology and Arrhythmology Clinic, Marche Polytechnic University, University Hospital ‘Ospedali Riuniti’, Ancona, Italy; Department of Neuroscience, Imaging and Clinical Sciences, G. d'Annunzio University of Chieti-Pescara, Chieti, Italy; Heart Department, University Cardiology Division, SS. Annunziata Hospital, Chieti, Italy; Department of Political Sciences, Communication and International Relations, University of Macerata, Macerata, Italy; Cardiology and Cardiovascular Pathophysiology, S. Maria della Misericordia Hospital, University of Perugia, Piazzale Giorgio Menghini, 3, Perugia 06129, Italy; Cardiology IV, ‘A. De Gasperis’ Department, Niguarda Ca’ Granda Hospital, Milan, Italy

**Figure 1:**
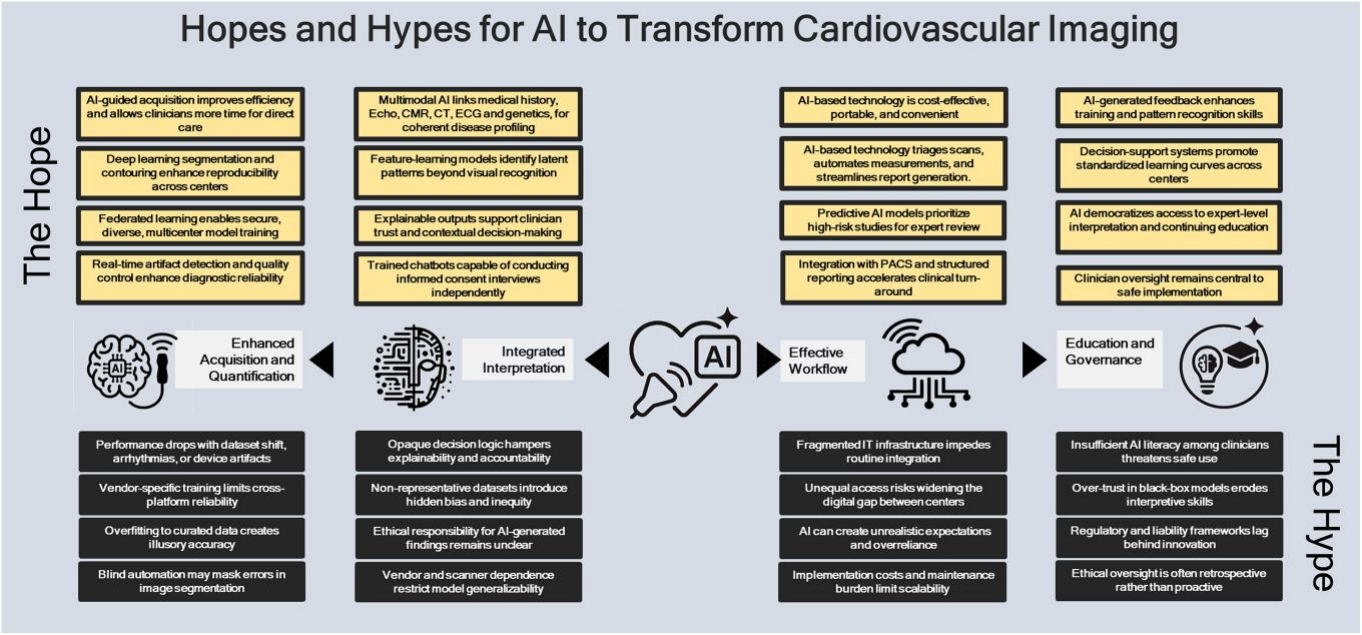
Hopes and hypes for artificial intelligence to transform cardiovascular imaging.

Artificial intelligence (AI) in cardiovascular imaging is not simply another technological evolution. Differently from prior social or technological innovations that impacted medicine, such as the use of Internet, AI impacted clinical practice immediately and not only research.^1^ The adoption happened extremely rapidly compared with the level of average operator experience available at the time of widespread implementation. The key reason is that AI provides very practical advantages across all cardiovascular imaging modalities, including transthoracic echocardiography, transoesophageal echocardiography, cardiac magnetic resonance, cardiac computed tomography, and nuclear cardiology^1,2^ (*Figure 1*). Among the most relevant applications already translated into clinical practice, the strongest impact is represented by automatic acquisition or guided acquisition,^3^ automatic segmentation, and automatic quantitative data extraction.^4–7^ This makes exams faster, reduces intra- and inter-observer variability, and generates large volumes of structured quantitative data that can improve phenotyping, diagnosis, risk stratification, downstream test selection, and therapy decision-making.^1^

However, these advantages cannot substitute expert human supervision (*Figure 1*). Cardiologists or radiologists must always perform qualitative assessment of all exams and must supervise segmentation and quantitative outputs. In these tasks, AI mistakes can originate from artefacts affecting segmentation or calculations, from arrhythmias such as atrial fibrillation with beat to beat variability, from complex multi-morbid conditions that require clinical reasoning, or from situations where quantitative interpretation cannot be contextualized without domain expertise.^2^ For example, although AI-based detection and severity assessment of aortic stenosis have been tested in large cohorts,^8^ performance remains insufficiently validated in complex scenarios involving significant aortic regurgitation, mitral regurgitation, restrictive cardiomyopathies such as cardiac amyloidosis, or severe diastolic dysfunction. In these cases, human correction and higher order clinical judgement remain still reasonably essential to prevent misclassification. In addition, errors may arise because the dataset used to train machine learning based algorithms is not representative of the current patient being assessed or of the environment where the exam is performed.^2^ Many current models originate from tertiary centres with highly curated data that do not reflect the majority of real-world centres. To overcome this limitation, datasets used to derive AI models should be as representative as possible of real-world populations and real-world resource settings. Federative data strategies should ideally include both tertiary and non-tertiary centres because non-tertiary settings represent, in the end, the majority of centres globally.

Another strategic frontier is integration (*Figure 1*). The future impact of AI will not only depend on single modality quantitative optimization, but on the capacity to integrate imaging data, clinical context, electrocardiogram data, laboratory biomarkers, and genetics.^1,7^ This has the potential to support differential diagnosis, to trigger targeted downstream testing, and to identify conditions that have strong implications for family screening and specific therapy. Fabry disease, an X-linked disorder caused by α-galactosidase A deficiency, exemplifies both the diagnostic complexity of rare cardiomyopathies and the transformative role AI could play. The diagnosis is often overlooked, particularly by clinicians unfamiliar with its heterogeneous cardiac manifestations. An integrated AI-enabled platform could generate the diagnostic hypothesis, guide enzyme and genetic testing, expedite referral to expert centres, and activate cascade family screening and timely initiation of therapy. A parallel can be drawn with cardiac amyloidosis, where a recent multicentre investigation demonstrated that a deep learning model applied to a single echocardiographic video clip, using only an apical four-chamber view, could reliably distinguish amyloidosis from other from other causes of increased left ventricular wall thickness.^9^ Together, these examples capture the emerging capacity of AI to bridge expertise gaps, transforming rare cardiomyopathies from diagnoses of exclusion into systematically recognizable and treatable diseases accessible to all levels of clinical practice. These advances exemplify how AI can extend subspecialty-level reasoning to routine practice, transforming rare cardiomyopathies from elusive diagnoses into systematically recognizable and treatable diseases.

There is also a long-term risk that must be explicitly addressed. Future generations of cardiologists may over rely on AI early in training for acquisition, reconstruction, and quantitative analysis, before they acquire the necessary independent skill to challenge AI errors (*Figure 1*). To prevent this, training programmes should initially limit AI use for decision substitution and instead deploy AI for education, pattern recognition learning, and simulation.^10^ Trainees must continue to learn classical qualitative and quantitative evaluation in order to develop their own judgement. AI should augment the formation of clinical reasoning, not shortcut it.

AI has transformed cardiovascular imaging more profoundly than any previous technological advance. Its impact derives from the direct improvement of acquisition, segmentation, and quantitative assessment, which naturally increases efficiency and standardization, while relying on the value of training datasets that accurately represent real-world populations and workflow. Yet, the responsible integration of AI requires vigilant human oversight, a solid grasp of algorithmic principles, awareness of inherent limitations, and rigorous grounding in clinical reasoning. Now the defining challenge is the pursuit of true harmonization wherein natural intelligence and clinical experience stand as the steadfast compass, and AI, as a learned companion, becomes the instrument that refines, amplifies, and extends their reach for the benefit of our patients.
